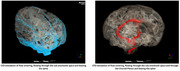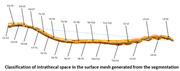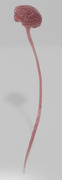# Computational Modeling and Simulation of the Cerebrospinal Flow and Drug Delivery

**DOI:** 10.1002/alz.094612

**Published:** 2025-01-09

**Authors:** Gustavo Montoya, Jaykumar Vishwanath Teli, Christopher Kadamus, Kathleen Lin, Kaitlyn Eddy, Eli Curry, Keith Blankenship, Nmachi Anumba, Rakesh Sharma, Vijay Kumar Sharma, Ujjal Bhanu Ghosh, Prasanth R, Carlos Corrales, Imran Sheikh, Dean Mariano, Christopher DeMaria, Kaushambi Roy, Rhea Sirkar, Joseph Katuin, Anand Subramony

**Affiliations:** ^1^ Eli Lilly and Company, Cambridge, MA USA; ^2^ Eli Lilly Services India Pvt., Bangalore India; ^3^ Eli Lilly and Company, Indianapolis, IN USA

## Abstract

**Background:**

The spinal intrathecal space, characterized by a complex three‐dimensional (3D) fluid‐filled geometry with varying levels of anatomic intricacy, plays a pivotal role in drug administration strategies targeting the central nervous system (CNS). Lumbar injections into this space represent a clinically used approach for delivering therapeutics directly to the brain, bypassing critical barriers such as the blood‐brain and blood‐cerebrospinal fluid barriers. A nuanced understanding of CSF dynamics is vital for comprehending physiological states of the CNS for drug delivery.

**Method:**

The CSF’s dynamic nature enables the dispersion of solutes throughout the brain and ventricular system. In order to optimize CSF drug delivery strategies, a profound comprehension of the intrathecal anatomy coupled with insights into the physiology of CSF generation, absorption, and flow, is imperative. The application of quantitative modeling tools, such as Computational Fluid Dynamics (CFD), yields critical insights into the impact of CSF fluid dynamics on drug dispersion and efficiency. CFD modeling provides a granular analysis of CSF flow field, surpassing the capabilities of Magnetic Resonance Imaging (MRI) or invasive methodologies.

**Result:**

Eli Lilly’s initiative in developing a CSF model aimed to predict drug distribution patterns mediated by cerebrospinal flow within the intrathecal space. This model sought to simulate prototypical CSF flow to ascertain the time required for a specified drug mass to reach the brain following lumbar intrathecal injection. Additionally, it aimed to assess the subsequent distribution of the drug within the brain, across a diverse patient demographic. This approach is instrumental in fine‐tuning drug delivery protocols, customizing delivery methods to particular drugs, avoiding off‐target drug effects, and ensuring maximum efficiency and efficacy for direct CNS treatments.

**Conclusion:**

Through this computational methodology, we have demonstrated the feasibility of obtaining validation data for the CFD simulations using several imaging methodologies and techniques. Furthermore, we have been able to build these complex models and use them to inform in vivo studies. We have also been able to demonstrate that it is possible to build comparable NHP and human models from the fluid dynamics perspective.